# Mesenchymal Stem Cell Therapy for Diabetic Kidney Disease: A Review of the Studies Using Syngeneic, Autologous, Allogeneic, and Xenogeneic Cells

**DOI:** 10.1155/2020/8833725

**Published:** 2020-11-20

**Authors:** Christian Sávio-Silva, Stephany Beyerstedt, Poliana E. Soinski-Sousa, Expedito B. Casaro, Maria Theresa A. Balby-Rocha, Antônio Simplício-Filho, Jamille Alves-Silva, Érika B. Rangel

**Affiliations:** ^1^Albert Einstein Research and Education Institute, Hospital Israelita Albert Einstein, São Paulo, SP, Brazil; ^2^Nephrology Division, Federal University of São Paulo, São Paulo, SP, Brazil

## Abstract

Diabetic kidney disease (DKD) is a microvascular complication of diabetes mellitus (DM) and comprises multifactorial pathophysiologic mechanisms. Despite current treatment, around 30-40% of individuals with type 1 and type 2 DM (DM1 and DM2) have progressive DKD, which is the most common cause of end-stage chronic kidney disease worldwide. Mesenchymal stem cell- (MSC-) based therapy has important biological and therapeutic implications for curtailing DKD progression. As a chronic disease, DM may impair MSC microenvironment, but there is compelling evidence that MSC derived from DM1 individuals maintain their cardinal properties, such as potency, secretion of trophic factors, and modulation of immune cells, so that both autologous and allogeneic MSCs are safe and effective. Conversely, MSCs derived from DM2 individuals are usually dysfunctional, exhibiting higher rates of senescence and apoptosis and a decrease in clonogenicity, proliferation, and angiogenesis potential. Therefore, more studies in humans are needed to reach a conclusion if autologous MSCs from DM2 individuals are effective for treatment of DM-related complications. Importantly, the bench to bedside pathway has been constructed in the last decade for assessing the therapeutic potential of MSCs in the DM setting. Laboratory research set the basis for establishing further translation research including preclinical development and proof of concept in model systems. Phase I clinical trials have evaluated the safety profile of MSC-based therapy in humans, and phase II clinical trials (proof of concept in trial participants) still need to answer important questions for treating DKD, yet metabolic control has already been documented. Therefore, randomized and controlled trials considering the source, optimal cell number, and route of delivery in DM patients are further required to advance MSC-based therapy. Future directions include strategies to reduce MSC heterogeneity, standardized protocols for isolation and expansion of those cells, and the development of well-designed large-scale trials to show significant efficacy during a long follow-up, mainly in individuals with DKD.

## 1. Introduction

### 1.1. Epidemiology

The global diabetes mellitus (DM) prevalence in 2019 was estimated at 9.3% (463 million) in adults aged 20-79 years, rising to 10.2% (578 million) by 2030 and 10.9% (700 million) by 2045 [1]. The prevalence is higher in urban (10.8%) than rural (7.2%) areas, and in high-income (10.4%) than low-income (4.0%) countries. Of importance, one in two (50.1%) people living with DM does not know that they have DM. Therefore, almost half a billion people are living with diabetes worldwide, and the number is projected to increase by 25% in 2030 and 51% in 2045. Likewise, the global prevalence of impaired glucose tolerance is estimated to be 7.5% (374 million) in 2019 and projected to reach 8.0% (454 million) by 2030 and 8.6% (548 million) by 2045 [1].

Using the WHO (World Health Organization) database, the International Diabetes Federation documented that 8.4% of all-cause deaths were attributable to DM in adults aged 20–79 years, almost 5.1 million deaths [2]. A sensitivity analysis adjusting relative risks by 20% found that the estimate of DM-attributable mortality lies between 5.1% of total mortality (3.3 million deaths) and 10.1% of total mortality (6.6 million deaths) [2]. Overall, 1 in 12 global all-cause deaths was estimated to be attributable to DM in adults [2].

Diabetic kidney disease (DKD) is a microvascular complication of DM and the most common cause of end-stage kidney disease (ESKD) worldwide, with approximately 30% of patients with type 1 DM (DM1) and approximately 40% of patients with type 2 DM (DM2) developing DKD, as reviewed elsewhere [3]. DKD accounts for cardiovascular complications and the high mortality rate of patients with DM. In the United States, the unadjusted prevalence of CKD stages 1-5 (not including ESKD) was estimated to be 14.8% (from 2011 through 2014), with stage 3 being the most prevalent stage [4]. There is an increase of 1.1% per year of new cases of ESKD, and the active waiting list is 2.8 times larger than the availability of donor kidneys.

### 1.2. Pathophysiology of DKD

Natural history of DKD comprises hyperfiltration, progressive albuminuria, decrease in eGFR (estimated glomerular filtration rate), and, ultimately, ESRD. Yet, albuminuria is a continuum; eGFR deterioration can start to decline before progression to overt nephropathy, which can be explained by other risk factors, such as obesity, hypertriglyceridaemia, hypertension, and glomerular hyperfiltration [5]. Thus, albuminuria and eGFR predict the progression of renal impairment in DM1 and DM2 individuals with DKD. Classification of DKD is summarized as follows: (i) stage 1 (prenephropathy): normoalbuminuria (<30 g/g Cr) and eGFR ≥ 30 ml/min/1.73 m^2^, (ii) stage 2 (incipient nephropathy): microalbuminuria (30-299 g/g Cr) and eGFR ≥ 30 ml/min/1.73 m^2^, (iii) stage 3 (overt proteinuria): macroalbuminuria (≥300 g/g Cr) or persistent proteinuria (≥0.5) and eGFR ≥ 30 ml/min/1.73 m^2^, (iv) stage 4 (kidney failure): any albuminuria status and eGFR < 30 ml/min/1.73 m^2^, and (v) stage 5 (renal replacement therapy): any status on continued dialysis therapy [6].

Histologically, metabolic changes associated with DM lead to glomerular hypertrophy, glomerulosclerosis, arteriolar hyalinosis, arteriosclerosis, tubule-interstitial inflammation, and fibrosis. The main glomerular changes consist of thickening of the glomerular basement membrane (GBM), expansion of the mesangial matrix, atrophy and loss of podocyte pedicels associated with effacement, and diffuse or nodular intercapillary glomerulosclerosis (Kimmelstiel-Wilson lesion) [3].

Systemic inflammatory milieu due to metabolic dysregulation (hyperglycemia, hyperlipidaemia, insulin resistance, and *β*-cell dysfunction) and haemodynamic changes (systemic hypertension) characterizes DKD pathophysiology. In addition, DKD is associated with endothelial dysfunction; activation of RAAS (renin-angiotensin-aldosterone system); increase in AGEs (advanced glycation end products); elevation of NADPH oxidase; upregulation of GLUT1; generation of reactive oxygen species (ROS); upregulation of growth factors, such as VEGF (vascular endothelial growth factor) and TGF-*β* (transforming growth factor-*β*); activation of aldose reductase and the polyol pathways; mitochondrial dysfunction; downregulation of adiponectin; and nitric oxide (NO) loss, as reviewed elsewhere [7, 8]. Those derangements entail adverse effects on the renal system, such as oxidative stress; apoptosis; autophagy dysfunction; intracellular signaling cascade activation, such as protein kinase C (PKC)/mitogen-associated protein kinase (MAPK) and subsequent NF-*κ*B; and inflammation, which is associated with inflammatory interleukins (IL), cytokines, and chemokines (IL-1, IL-6, IL-18, TNF-*α* (tumor necrosis factor-*α*), CSF-1 (colony stimulating factor-1), MCP-1 (monocyte chemoattractant protein-1), and MIF (macrophage inflammatory factor)). Exacerbated production of profibrotic cytokines (CTGF (connective tissue growth factor) and TGF-*β*) associated with fibrosis is also involved in DKD. Collectively, all those mechanisms contribute to DKD progression and to both functional (declining eGFR and proteinuria) and structural (fibroblast accumulation, mesangial cell expansion and proliferation, extracellular matrix accumulation, GBM thickening, podocyte loss/dysfunction, tubule-interstitial dysfunction, and endothelial dysfunction) kidney damage, which lead ultimately to systemic complications (ESKD, cardiocerebrovascular events, vascular events, neuropathy, and death).

### 1.3. Treatment

Due to DM prevalence worldwide, it is crucial to develop cost-effective strategies at every step: (1) prevention of obesity, (2) screening for and prevention of diabetes in an at-risk population, (3) glycemic control once diabetes develops, (4) blood pressure (BP) control once hypertension develops, (5) screening for diabetic chronic kidney disease (CKD), (6) RAAS inhibition/blockade in those with diabetic CKD, and (7) control of other cardiovascular (CV) risk factors such as management of low-density lipoprotein cholesterol (LDL-C) [9, 10].

Despite diabetic patients being treated with angiotensin-receptor-blockers (ARBs), renal disease progression risk over 2 years increases with increasing proteinuria and albuminuria and decreasing eGFR [11]. To note, RAAS inhibition possesses remarkable renoprotective effect when used in earlier stages of renal disease, whereas in late stages, that approach has less efficacy [12]. Yet, the combination of ARBs and angiotensin-converting-enzyme (ACE) inhibitors is a robust approach to block RAAS; it was associated with an increased risk of adverse events, such as acute kidney injury and hyperkalemia [13].

Novel drugs have been recently associated with clinical benefit profiles, which should be considered in the decision-making process when treating patients with DM2. Glucagon-like peptide 1 receptor agonists (GLP1-RA) and sodium-glucose cotransporter-2 inhibitors (SGLT2i) reduce atherosclerotic major adverse cardiovascular (CVs) events to a similar degree in patients with established atherosclerotic CV disease, whereas SGLT2i have a more marked effect on preventing hospitalization for heart failure and progression of DKD [14, 15].

In the DKD treatment setting with drugs and lifestyle changes, novel approaches are further required to halt the progression of DKD or regenerate the damaged tissue, such as cell therapy [16]. In this review, we will focus on both *in vitro* and *in vivo* studies using syngeneic, autologous, allogeneic, or xenogeneic mesenchymal stem cells (MSCs) for treating DKD. We will describe the main findings of MSC-based therapy in preclinical and clinical studies and discuss the benefits, outcomes, and challenges of that therapy for halting DKD progression.

## 2. Mesenchymal Stem Cells (MSCs)

MSCs, commonly referred to as mesenchymal stem cells or mesenchymal stromal cells, are a diverse population of cells with a wide range of potential therapeutic applications for different organs and tissues. MSCs can be obtained from many tissue sources, consistent with their broad, possibly ubiquitous distribution.

Historically, MSCs were isolated from bone marrow (BM-MSC) and spleen from guinea pigs by Friedenstein et al. [17]. They observed that BM-MSCs were plastic adherent cells and were capable of forming single-cell colonies. When BM-MSCs are expanded in culture, round-shaped colonies resembling fibroblastic cells are formed and subsequently identified by a Colony Forming Unit-fibroblast (CFU-f) assay. They were the first to demonstrate that BM-MSCs exhibited multipotential capacity to differentiate into mesoderm-derived tissues.

BM-MSCs can be isolated by (a) using gradient centrifugation (Ficoll or Percoll) to separate nonnucleated red blood cells from nucleated cells, (b) taking advantage of their ability to adhere to plastic, (c) taking advantage of the ability of monocytes to be separated from BM-MSCs by trypsinization [18].

During the 1980s, BM-MSCs were found to be able to differentiate into osteoblasts, chondrocytes, adipocytes, and muscle tissue [19]. In the 1990s, BM-MSCs were shown to differentiate into ectodermal-derived tissue [20, 21]. During the early 21^st^ century, *in vivo* studies documented that human BM-MSCs differentiated into endodermal-derived cells [22, 23], cardiomyocytes [24], and renal mesangial and epithelial tubular cells [25, 26]. However, their efficiency to differentiate into other tissues is extremely low *in vivo* and therefore is not the main mechanism of tissue repair and regeneration.

More recently, BM-MSC secretome has demonstrated potential clinical applications and includes both soluble proteins (cytokines, chemokines, growth factors, and proteases) and factors released in extracellular vesicles, for example, microvesicles (size 100-1000 nm) and exosomes (EXOs; size 40-100 nm) [27]. These extracellular vesicles contain proteins, lipids, mRNA, and miRNA and rarely DNA [28]. Mitochondria or mitochondrial DNA can also be transferred by extracellular vesicles or nanotubes built between cells that are regulated by dynamin-related proteins Miro-1 and Miro-2 [29]. Therefore, BM-MSC secretome is involved in cell survival and growth, immune modulation, and attenuation of fibrosis. High-resolution proteomic and lipidomic analyses have shown that key regulators of some pathways are enriched in both microvesicles and EXOs, including GTPase activity, translation, vesicle/membrane, and glycolysis, whereas other pathways are enriched more in microvesicles (cell motion, mitochondria, endoplasmic reticulum, and proteasome) and others in EXOs (extracellular matrix, binding, immune response, and cell adhesion) [30].

Of importance, MSCs possess ubiquitous distribution in perivascular niches and can be derived and propagated *in vitro* from different organs and tissues (AT, amniotic fluid, BM, brain, cord blood, dental pulp, kidney, liver, lung, muscle, pancreas, placental membranes, spleen, thymus, and large vessels, such as aorta artery and vena cava) [31, 32]. Most frequent sources of MSC isolation include BM, adipose tissue (AT-MSC), and umbilical cord blood (UCB-MSC). In BM, one in 10,000 nucleated cells is a MSC. To note, 1.0 g of aspirated AT yields approximately 3.5 × 10^5^‐1 × 10^6^ AT-MSCs. This is compared to 5 × 10^2^‐5 × 10^4^ of BM-MSCs isolated from 1.0 g of BM aspirate [33].

Isolation of MSCs from AT is based on mincing fat tissue, followed by several washings in order to remove contaminating hematopoietic cells, incubation of tissue fragments with collagenase, and centrifugation of the digest, thereby separating the floating population of mature adipocytes from the pelleted stromal vascular fraction [34]. UCB-MSC is also a straightforward protocol and consists in carefully dissecting the UC into two regions, e.g., the cord lining and Wharton's jelly. After cutting the UCB longitudinally, it is necessary to scrape Wharton's jelly away from the blood vessels and inner epithelium and then remove the blood vessels. After collecting any remaining perivascular Wharton's jelly tissue under and around the blood vessels, which represents the cord lining, the digestion of that tissue with trypsin will allow the adherence of tissue pieces and the egression of MSCs in 2-3 days, as briefly described elsewhere [35].

MSC populations originating from different tissues and organs exhibit similar morphology and, to a certain extent, surface marker profile [31]. On the other hand, differentiation assays indicate some variation among cultures in the frequency of cells that possess the capacity to differentiate into osteogenic or adipogenic lineages. For example, vena cava-derived MSCs were very efficient at depositing a mineralized matrix, whereas muscle-derived MSCs showed little efficiency for osteogenic differentiation, as opposed to an inverse capacity of adipocyte differentiation of these cells [31]. Conversely, adipogenic differentiation observed in lung-, brain-, and kidney-derived MSCs seemed to be less efficient. Likewise, UCB-MSCs exhibit significantly stronger osteogenic capacity but lower capacity for adipogenic differentiation in comparison to BM-MSCs [36]. Of importance, AT-MSCs exhibit similar capacity of differentiation when compared to BM-MSCs [37].

The International Society for Cell Therapy (ISCT) established the characteristics of MSCs from all sources, either autologous or allogeneic: (1) adherence to plastic under standard culture conditions; (2) expression of CD73, CD90, and CD105 surface molecules in the absence of CD34, CD45, HLA-DR, CD14 or CD11b, CD79, or CD19 surface molecules, as assessed by flow cytometry analysis; and (3) differentiation capacity for osteoblasts, adipocytes, and chondroblasts *in vitro* [38]. In comparison to fibroblasts, both cells express CD44 and CD49b, whereas CD20, CD31, CD33, CD117, and CD133 are negative in both cells. Some markers are only expressed in MSCs (CD10, CD26, CD54, CD106, CD146, and ITGA11), as well as the potential of colony forming [39].

A recent update from ISCT includes analyses that mitigate the heterogeneity of MSCs, such as assays that demonstrate the secretion of trophic factors, the modulation of immune cells, and other relevant functional properties, such as angiogenesis [40]. The ISCT MSC committee recommended that the studies should describe (i) tissue source origin of MSCs, which would highlight tissue-specific properties; (ii) the stemness properties described by both *in vitro* and *in vivo* data; and (iii) a robust matrix of functional assays to demonstrate the properties of these cells associated with the intended therapeutic mode of actions. In addition, basic assays for MSC-based products comprise donor screening, viability test, purity test (residual contaminant tests and pyrogenic/endotoxin tests), safety test (bacterial, fungal, mycoplasma, viral tests, and tumorigenicity assays), identity tests (immunophenotypic profiles), and potency tests (multilineage differentiation, secretion profiles, CFU-f assay, and immunosuppressive assay). All of these procedures should be done in a Good Manufacturing Practice (GMP) facility.

To assess MSC self-renewal capacity, doubling time and CFU-f are broadly used. In a particular DM setting, immunological assays can be based on activation protocols that discriminate between TLR- (Toll-like receptor-) 4-dependent phenotype MSC-1 and TLR3-dependent MSC-2 phenotype [41]. That polarization may be achieved with short-term incubation (1 h) with LPS (10 ng/ml) or poly(I:C) (poly-deoxy-inosinic-deoxy-cytidylic acid) (1 mg/ml), respectively, followed by incubation for 24 to 48 h in growth medium, since LPS acts as an agonist for TLR4 and poly(I:C) acts as an agonist for TLR3. Another approach for assessing MSC-based immunomodulatory properties would be based on the coculture of MSCs with cells of the immune system by the (a) stimulation of MSCs with IFN-*γ* (IFN-*γ* primed MSCs) and subsequent analysis of various ribonucleic acids (IDO, CXCL9, CXCL10, CXCL11, CIITA, HLAD, and PDL1 or CD274, ICAM-1 or CD54, TLR3, TRAIL, and CCL5) and (b) coculture of MSCs with human peripheral blood mononuclear cells (PBMCs) and analysis of the signature of the secretome in relation to cytokine/chemokine secretion and T cell proliferation [42]. Coculture of MSCs and B-lymphocytes and NK cells may also be a useful strategy to assess MSC-based immunomodulatory properties. To note, such assays are important in addressing MSCs before and after freezing. It is also worth mentioning that PBMCs should be used from donors that show a normal pattern of proliferation and without much variability.

### 2.1. *In Vitro* Studies: Recapitulation of DKD Microenvironment for Evaluating the Therapeutic Potential of MSCs

To recapitulate, the *in vitro* DKD milieu is challenging since cell-cell and cell-matrix interaction is severely affected during disease progression. The most frequent approach is to evaluate the cells under normal glucose medium (5.5 mmol/l) and high glucose (25-30 mmol/l or less frequent 40 mmol/l). Mannitol (20 mmol/l) associated with normal glucose (5.5 mmol/l) is used as a control of osmolality. Peroxide hydrogen and TNF-*α* may be added to the medium as inducers of oxidative stress [43] and inflammation [44], respectively. The coculture of MSCs, MSC-conditioned medium or EXOs, and different types of renal cells represents a platform in which the DKD microenvironment may be recapitulated. The most appropriate approach to recreate DKD *in vitro* (high glucose, peroxide hydrogen, and TNF-*α*), the amount of cells (ratio of MSCs and renal cells), the type of cell interaction (direct versus indirect, e.g., using a Transwell® chamber), and duration of the coculture (6 h, 12 h, 24 h, 48 h, 72 h, or 96 h) were broadly tested in the literature.

Immortalized mouse podocytes cultured in high glucose medium and cocultured directly with BM-MSC transfected with miR124a, for 24 h, exhibited increased viability and decreased apoptosis (decrease in caspase-3 and Bax gene expression and increase in Bcl2 gene expression) [45]. Mouse podocytes (MPC5 cells) treated with high glucose medium and cocultured with AT-MSC-derived EXOs, for 24 h, 48 h, 72 h, and 96 h, exhibited less apoptosis in concentration- and time-dependent manners [46]. Mechanistically, AT-MSC-derived EXOs enhanced autophagy flux and reduced podocyte injury by inhibiting the activation of mTOR/SMAD1 signaling and increasing miR-486 expression.

For glomerular mesangial cells (GMCs) cultured in high glucose medium, direct coculture with BM-MSC (ratio 10 : 1) or MSC-conditioned medium for 72 h decreased equally TGF-*β* and phosphorylated SMAD2/3 proteins, which were abrogated by BMP-7 antibody [47]. Likewise, GMC cultured in a high glucose medium and cocultured with BM-MSC (4 × 10^5^ cells/well) in a Transwell® chamber for 72 h led to an increase in lipoxin A4, a key lipid involved in inflammation resolution [48].

For renal tubular epithelial cells (TECs) cultured in high glucose medium, the coculture for 24 h with AT-MSC (1 × 10^5^ cells/well) using a Transwell® chamber inhibited apoptosis of those cells, induced klotho expression, and downregulated the Wnt/*β*-catenin signaling pathway [49]. In addition, high glucose medium supplemented with TNF-*α* may also mimic the DKD microenvironment [44]. In that study, proximal TECs (HK2) were cocultured with UCB-MSC in a Transwell® chamber at a 5 : 1 ratio, for 72 h, in high glucose medium and TNF-*α*. UCB-MSC increased cell viability, ATP production, and E-cadherin expression, as opposed to a decrease in fibronectin, SGLT2, pNF-*κ*B p65, and MCP-1.

Not only MSCs but also EXOs cocultured for 96 h with TECs in primary renal cell culture of streptozotocin- (STZ-) induced diabetic rats entailed in antiapoptotic and antidegenerative effects (increase in ZO-1 and lectin expression and decrease in TGF-*β*1) [50].

Endothelium may also be damaged during DM progression. Thus, the murine islet microvascular endothelium cell line experienced apoptosis and endothelial cell activation (increase in VCAM (vascular cell adhesion molecule) expression and reduction in eNOS (endothelial nitric oxide synthase) phosphorylation) upon H_2_O_2_ conditioning, which was abrogated by MSC treatment and activation of the *β*-catenin-dependent Wnt signaling pathway [51].

Therapeutic potential of MSCs can also be verified in a coculture platform with other cells that play a role in DKD progression, such as macrophages. Indirect coculture of BM-MSC (3 × 10^4^ cells/well) with LPS-treated macrophages (rat peritoneum; 1.5 × 10^5^ cells/well), at a 1 : 5 ratio for 6 h, led to a decrease in IL-1*β*, IL-6, MCP-1, and TNF-*α* expression [52]. Coculture of immortalized macrophage cell line (RAW264.7) with human UBC-MSCs (at a 2 : 1 ratio), for 24 h, suppressed LPS-induced M1 macrophage polarization (decrease in inflammatory proteins, such as IL-1*β*, TNF-*α*, IL-6, and iNOS (inducible nitric oxide synthase)), which was mediated by the increase in arginase 1 production [53]. To note, iNOS metabolizes arginine to nitric oxide and citrulline, whereas arginase (M2-macrophage) hydrolyzes arginine to ornithine and urea. Therefore, the arginase pathway limits arginine availability for nitric oxide synthesis, and ornithine itself can further lead to polyamine and proline synthesis, which have important biological implications for proliferation and tissue repair. In addition, MSC-conditioned medium reversed cytokine-mediated mitochondrial dysfunction in HK2 cells (TECs) by increasing mitochondria mass and biogenesis and decreasing ROS production [53].

Aging has also an adverse impact on MSC function and possesses biological and therapeutic implications. Moreover, CKD and DM are linked to accelerated aging. The p66 protein is related to aging and controls cellular response to oxidative stress, senescence, and apoptosis. Renal-derived Sca-1^+^ MSCs from p66 knockout mice and cultured in high glucose medium exhibited higher rates of proliferation; decreased senescent proteins (p53, p21, and p16^INK4a^); higher levels of IGF-1 (insulin growth factor-1), HGF (hepatocyte growth factor), and VEGF; and upregulation of *β*-catenin signaling when compared to renal-derived Sca-1^+^ MSC from wild-type mice [54].

### 2.2. Preclinical Studies: Small and Large Animals

MSC-based therapy is a promising strategy for accelerating kidney recovery, repairing and regenerating tissue damage after acute injury following ischemia-reperfusion, kidney transplant, and drug-mediated toxicity, as reviewed elsewhere [55]. In a meta-analysis including MSC from rat and mice (~200 animals treated) and different types of acute and chronic kidney injury (but not DKD), routes of delivery (intravenous, intrarenal, intraperitoneal, and intra-arterial), and MSC number (range, 7.5 × 10^4^‐3.0 × 10^6^), the beneficial outcomes for kidney recovery favored MSC treatment [56].

Of importance, MSC efficacy is challenged by several factors, such as viability, cell source, MSC phenotype, homing capacity, route of delivery, site of infusion, number of infusions, cell passage, cell potency, severity of condition, and target impact [57]. In the sensitivity analysis of that meta-analysis, there was a trend toward greater reduction in serum creatinine of the MSC-treated group when compared with the control group regarding the MSC number (>106), arterial route (versus intravenous route), model of injury (ischemia-reperfusion injury versus toxic and chronic injury), and late administration (>1 day after injury) [56]. Thus, these data provided insightful information in terms of MSC efficacy and safety in preclinical models and paved the way for studies in other kidney diseases, such as DKD.

Next, we discuss some key aspects of MSC-based cell therapy in preclinical studies.

#### 2.2.1. MSC Phenotype

Emerging concepts indicate that MSCs may function as sensors and switchers of inflammation, which may explain their immunomodulatory properties [58, 59]. In an inflammatory environment associated with high levels of IFN-*γ* (interferon-*γ*) and TNF-*α*, MSCs acquire an immunosuppressive phenotype (MSC2) and through Toll-like receptor- (TLR-) 3 lead to an increase in production of TGF-*β*, IDO (indoleamine 2,3-dioxygenase), NO (nitric oxide), and PGE2 (prostaglandin E2). These events stimulate the amount of CD4^+^CD25^+^FoxP3^+^ T regulatory cells. Conversely, in the absence of an inflammatory environment (low levels of IFN-*γ* and TNF-*α*), MSCs acquire a proinflammatory phenotype, and through TLR4, LPS (lipopolysaccharide), and high levels of chemokine C-X-C motif ligand (CXCL)9, CXCL10, MIP- (macrophage inflammatory protein-) 1*α*, MIP-1*β*, and CCL5/RANTES (regulated on activation, normal T cell expressed and secreted), but low levels of IDO, NO, and PGE2, activation of cytotoxic T lymphocytes is triggered.

Interaction of MSCs and monocytes play also a key role in our understanding of mechanisms of MSC-mediated tissue regeneration [58, 59]. When MSCs acquire an immunosuppressive phenotype (high levels of IDO and PGE2) in the presence of IL-6, there is a polarization from monocytes (M0) to macrophage anti-inflammatory phenotype (M2 macrophages; CD206 and CD163 expression; production of high levels of IL-6 and IL-10). On the other hand, proinflammatory MSC-induced phenotype may lead to polarization from M0 to proinflammatory macrophage (M1 macrophage; CD86 expression; production of high levels of IFN-*γ* and TNF-*α*).

However, further investigation is warranted to verify whether MSC phenotype changes in accordance with DKD progression. In other settings, such as kidney transplant, MSC infusion posttransplant allowed their preferential recruitment in the inflammatory milieu of the graft created by ischemia/reperfusion injury, and once in that environment, MSC contributed to upregulation of inflammation, thereby causing premature graft dysfunction [60]. By contrast, autologous BM-derived MSC infusion induced a significant prolongation of kidney graft survival by a T cell regulatory-dependent mechanism when a protocol biopsy showed signs of subclinical rejection and/or an increase in interstitial fibrosis/tubular atrophy 4 weeks or 6 months posttransplantation [61]. Additionally, autologous BM-derived MSC, when injected before living-related kidney transplant, led to a decrease in the circulating memory CD8^+^ T lymphocytes and donor-specific CD8^+^ T lymphocyte cytolytic response [62] and might induce tolerance [63].

#### 2.2.2. Routes of MSC Delivery

Stem cell route delivery (intravenous, intra-arterial, or intraparenchymal) may affect MSC efficiency for kidney repair and regeneration in different models of acute and chronic kidney injury. The intravenous route is the route used most often, to inject not only MSCs [64–67] but also different kidney-derived progenitor/stem cells [68, 69] in several models of acute and chronic kidney injury in rodents. To note, MSCs, BM-derived mononuclear cells (BM-MNCs), and other kidney progenitors are initially trapped inside the pulmonary microvasculature following intravenous administration [70]. In line with these findings, the number of cells, multiple intravenous injections, and cell size increase the chance of pulmonary trapping, as murine MSCs measure 15-19 *μ*m [70, 71]. Similar observations were reported in nonhuman primates when MSCs were injected intravenously [72, 73]. Sodium nitroprusside pretreatment, a vasodilator, may reduce mouse MSC trapping in the lungs [71] and require further analyses of its efficiency in larger animals.

However, infused human MSCs are able to migrate beyond the lungs after intravenous administration in a rodent model of cisplatin-induced acute kidney injury and may be detected in peritubular areas, where they ameliorated renal cell apoptosis and increased cell proliferation [74].

Intra-arterial routes for delivering progenitor/stem cells include intracarotid [75], intracardiac [76], or intra-aorta [77–81]. When the intra-aorta route is employed, the clamps can be applied above and below the renal arteries [77, 78] or only below the renal arteries [79–81], which can be challenging in small animals [82]. Bioluminescence analyses supported a distinct localization of MSCs in the murine kidneys submitted to ischemia-reperfusion injury when these cells were injected in the suprarenal aorta (intracarotid), in contrast to intrajugular vein injection, which was associated with predominant accumulation of cells in both lungs [83]. In larger animals (ovine), autologous MSCs delivered through renal arteries were also effective in reducing tubular injury after ischemia-reperfusion injury [84].

Although intraparenchymal (under renal capsule) administration of progenitor/stem cells or MSCs has beneficial effect on kidney repair [79, 85–89], this route is less practical for clinical application, especially when the renal disease is diffuse and technical issues limit a broader use, such as haemorrhage. However, the bioengineering field has undergone considerable evolution, so that MSC sheets may be transplanted directly into the kidneys and suppress the progression of DKD in rats [90].

#### 2.2.3. MSC Homing (CXCR4 and SDF-1 Axis)

Stromal-derived factor-1 (SDF-1), also known as CXCL12, and its receptor C-X-C chemokine receptor 4 (CXCR4) axis is a crucial key pathway in cell trafficking.

After acute kidney injury, the levels of SDF-1 mRNA levels increase more than 2.5-fold and remain high as ~2.0-fold after 24 h within kidney cortex tissue [91]. That increase leads to homing and migration of CXCR4-expressing cells in the injured kidneys. However, MSCs, which express CXCR4, migrate to damaged tissues with limited efficiency. Therefore, CXCR4 gene-modified BM-MSCs lead to accumulation of these cells in the injured kidney and activation of PI3K/AKT and MAPK signaling pathway [92], which represents a promising strategy for advancing MSC-based therapy.

#### 2.2.4. Animal Models of Diabetic Kidney Disease

There are several animal models of DKD in rodents, which mimics DM in humans either DM1 or DM2. Therefore, DM and subsequent DKD can be obtained by genetic manipulation, induced by drugs (streptozotocin or STZ) or high-fat diet, or even a combination of approaches, including uninephrectomy to accelerate DKD progression, as reviewed elsewhere [16].

Thus, pharmacologic induction of DKD with STZ, with or without accelerating factors, such as high-fat diet, uninephrectomy, or use of the nonobese diabetic (NOD) strain, has been the most common rodent model of DKD to study the potential therapy of MSCs [7].

The Animal Models of Diabetic Complications Consortium (AMDCC) defined the following criteria for validating a progressive mouse model of DKD [93]: (i) greater than 50% decline in GFR over the lifetime of the animal; (ii) greater than 10-fold increase in albuminuria compared to controls for the strain at the same age and gender; and (iii) kidney-specific histopathology induced by DM: advanced mesangial matrix expansion ± nodular sclerosis and mesangiolysis, any degree of arteriolar hyalinosis, and GBM thickening by >50% over baseline tubule-interstitial fibrosis.

Recent models of DM1 (E1-DKD; expression of a kinase-negative epidermal growth factor receptor in pancreatic islet cells e) and DM2 (BTBR*^ob/ob^*; knockout for leptin) that reflect human DKD [94, 95] may represent promising models to verify not only stem cell-based therapy but also drug, gene, nanoparticle, and other approaches to halt DKD progression [16]. E1-DKD and BTBR*^ob/ob^* models develop proteinuria in a time-dependent manner, mesangial expansion, thickening of GBM, widening of podocyte foot process, podocyte apoptosis, glomerular sclerosis, and reduction of podocyte genes and protein. Notably, BTBR*^ob/ob^* mice comprise a reversible model of DM upon leptin administration [96], which indicates, therefore, a robust model to test MSC therapeutic potential.

NOD mice develop autoimmune insulitis caused by polygenes including specific MHC class II alleles and many non-MHC loci, mimicking DM1 [97]. NOD mice develop albuminuria associated with enlarged glomeruli and mesangial sclerosis. An insulin-2 Akita mouse exhibits an autosomal dominant mutation in the Ins-2 gene that causes misfolding of insulin protein [97]. These mice develop increased mesangial matrix and GBM thickening, but no mesangiolysis or widespread marked or nodular mesangial sclerosis. Similarly, the *db/db* mouse is a model of DM2, which develops hyperglycemia, obesity, and albuminuria due to a G-to-T mutation in the gene coding the leptin receptor (*db/db*) [97]. They develop glomerular hypertrophy, mesangial matrix expansion, and GBM thickening, but no mesangiolysis or nodular mesangial sclerosis. The Otsuka Long-Evans Tokushima Fatty (OLETF) rat model of hyperphagia-induced obesity due to a spontaneous lack of CCK_1_ (cholecystokinin) receptors represents a broadly established model of DM2, which develops proliferation of the mesangial matrix, GBM thickening, diffuse glomerulosclerosis, nodular lesions, tubular atrophy associated with mononuclear cell infiltration, and fibrosis [97]. Other rodent models of DM2 and DKD include GK rat, NZO mouse, KK-Ay mouse, and ZDF rat, as reviewed elsewhere [97].

In Table 1, we document the preclinical studies, including the MSC source, number of cells and injections, route of delivery, and outcomes in the DKD setting [44, 45, 47, 48, 49, 50, 52, 90, 98–118]. The majority of the studies comprised syngeneic MSCs obtained from BM, single-dose injection via an intravenous route, and successful outcomes for halting DKD progression.

Briefly, these studies provided evidences that MSC-based therapy may decrease fasting blood glucose (FBG) and glycated haemoglobin (HbA1c) in either DM1 or DM2 animals, and in DM1 animals, plasmatic insulin levels increased or exogenous insulin requirement decreased. Likewise, MSC-based therapy has important therapeutic implications in the DKD setting, providing insights into cellular and molecular mechanisms. Therefore, MSCs contributed to improving functional parameters, such as the increase in glomerular filtration and the decrease in albuminuria and structural parameters. The studies indicated an improvement in renal histology and the curtailing of biological processes of inflammation, cell death (apoptosis and necrosis), oxidative stress, and fibrosis. In addition, MSC-based therapy promoted preservation of renal mass, upregulation of tubular epithelial and podocyte genes, augmentation in growth factors within the kidneys, decreasing endothelium damage, amelioration of tubular glucotoxicity by decreasing cellular glucose uptake in the kidneys, and increasing the antiaging klotho protein.

Differentiated BM-MSCs to insulin-secreting *β*-cells may also represent a promising strategy to treat DM and clinical complications, as documented by the amelioration of endothelium activation by decreasing fibrinogen levels, blood pressure, cytoplasmic calcium, and apoptosis (p53 and Bax), as well as by improving cardiac parameters in STZ-induced diabetic rats [119].

Likewise, secretome of BM-MSC obtained from Zucker DM2 fatty rats improved endothelial cell function by increasing ~3-fold the formation of tubule-like structures and migration of these cells, which was mediated by IGF-1, LTBP-1 (latent TGF-*β* binding protein), and LTBP-2, as well as by promoting vascular formation *in vivo* [120]. In addition, diabetic secretome exhibited increased expression of proangiogenic genes (ANPEP, MCP-1, MIP-2, HIF-2, IGF-1, IL-6, PLAU, TIE1, and TNF-*α*) and reduced antiangiogenic genes (COL18A1, COL4A3, F2, IFN-*γ*, and TGF-*β*1/3). Extracellular matrix-related proteins (FMOD, OSTP, and COBA1) were also higher in diabetic secretome. These data indicate that BM-MSCs from DM2 rats have a unique secretome with distinct angiogenic properties and provide new insights into the role of BM-MSCs in aberrant angiogenesis in the diabetic milieu.

The hyperglycemic milieu may also adversely impact MSC functionality. Therefore, AT-MSC extracted from Zucker diabetic fatty rats exhibited downregulation of markers of pluripotency (lower capacity of osteogenic and endothelial differentiation *in vitro*) and self-renewal, which may compromise the efficiency of direct self-repair and autologous cell therapy [121]. In addition, these cells exhibited loss of viability, impairment of capillary-like tube formation in Matrigel, decreased expression of stemness genes, signaling pathways important for stem cell maintenance (Nocth1, Notch2, Wnt1, and Dhh) genes, and cell trafficking (CXCL2 and CXCR4) genes, as well as decreased angiogenesis *in vivo* [121].

Likewise, MSCs extracted from rodents with DM2 or large animals with metabolic syndrome have morphological abnormalities (larger number of degenerated mitochondria and marked expansion of endoplasmic reticulum), less proliferative potential associated with an increase in doubling time, alteration in gene expression (downregulation of growth factors IGF-1 and EGF, and angiogenic factors TBX1 and TBX5, and upregulation of proinflammatory genes IFN-*γ* and IL-1*β*. IL-2, regulated on activation normal T cell expressed and secreted (RANTES), TNF-*α*, as well as alpha muscle actin, which represents the stress fiber, and XBP-1, which represents endoplasmic reticulum stress), greater senescence, lower viability and homing capacity, increased apoptosis, and a reduction in clonogenic and multidifferentiation potentials [115, 122, 123]. Conversely, BM-MSCs from diabetic rodents may preserve their multipotent capacity when compared to nondiabetic animals [124].

Of importance, studies with longer duration are required to improve our understanding on the safety profile of MSC-based therapy, such as the cytogenetic aberrations observed during the propagation of these cells in culture. In MSCs derived from mice (C57BL/6 and BALB/c), such aberrations were observed after several passages *in vitro* [125], as well as their malignant transformation *in vivo*, either after injection [126] or promoting the growth of a preexisting tumor [127]. The injection of human (xenogeneic) MSCs in murine models may be associated with the formation of tumors in these animals, as well as with other structural changes, such as chronic jejunitis and villous atrophy, during a three-month follow-up period [128].

### 2.3. Autologous-Derived MSC for Halting the Progression of DKD in Humans: Advantages and Drawbacks

BM-MSCs are the main source of autologous cell transplantation for various diseases including DM-related micro- and macrovascular complications [129]. Therapy with autologous MSCs is of great interest and has advantages for the patient, as these cells are readily available. MSC-based therapy is based on the extraction of these cells from the patient, expansion *in vitro*, and injection back into the patient, thus avoiding complications resulting from graft rejection and/or the need for an immunosuppressive regimen. Therefore, while patient-derived (autologous) MSC may be the safer choice in terms of avoiding unwanted immune response, factors including donor comorbidities (DM, chronic kidney disease, hypertension, and others) and aging may preclude those cells from use.

Notwithstanding recent promising results with MSC therapy in several diseases, moving the concept forward toward the DKD setting should be critically assessed by looking for intrinsic MSC abnormalities caused by the hyperglycemic milieu, which may adversely affect their therapeutic potential in diabetic patients. Thus, AT-MSCs extracted from diabetic individuals have a greater capacity for adipogenic differentiation, but less chondrogenic and osteogenic differentiation [130, 131]. Conversely, BM-MSCs from diabetic individuals preserve their multipotent capacity [113]. Therefore, the source of MSC may play a critical role in decision-making for treating diabetic individuals. AT-MSCs isolated from the ischemic limb of diabetic patients seem to be less potent when compared phenotypically and functionally to control nondiabetic counterparts with no signs of limb ischemia [132]. To note, 40% of diabetic and 20% of nondiabetic AT-MSC samples displayed high expressions of fibroblast marker, which inversely correlated with the expression of CD105. In diabetic patients, significantly decreased expression of VEGF and CXCR4 was verified in fibroblast-positive AT-MSCs when compared to their fibroblast-negative counterparts, which may negatively affect angiogenic and homing capacity mediated by AT-MSCs, respectively [132]. Reduced osteogenic differentiation and the downregulation of chemokine CXCL12 were also observed in fibroblast-negative diabetic AT-MSCs. Both diabetic and nondiabetic AT-MSCs were able to differentiate into adipocytes and chondrocytes, yet not exhibiting islet-like cell differentiation in that study [132]. Importantly, *in vitro* studies documented the differentiation potential of human AT-MSCs into islet-like cells when these cells were obtained from healthy individuals who underwent abdominoplasty or liposuction [133–135]. Transdifferentiated cells exhibit positive staining for dithizone, increased expression of islet cell-related genes (Pdx-1, Isl1, Ngn3, NeuroD1, Pax4, and GLUT2), and insulin secretion when these cells were challenged with high concentrations of glucose.

Not only the source of MSCs but also the type of DM may affect the therapeutic potential of MSCs. MSCs extracted from DM1 individuals exhibited preserved morphology, growth kinetics, multipotency, and proliferative, immunomodulatory, immunosuppressive, and migratory capacities [113, 136].

In contrast, MSCs extracted from individuals with DM2 have greater senescence, lower viability, increased apoptosis (increased proapoptotic gene expression, such as p53, caspase 9, and BAX, and low antiapoptotic gene expression, such as Bcl-2), less proliferative potential associated with increased doubling time, and a reduction in angiogenic potential [130, 137].

CD105 (endoglin) is associated with angiogenesis [138], and its positivity in AT-MSC leads to higher rates of proliferation [139]. Therefore, reduced CD105 expression and proliferation of AT-MSC in DM2 individuals indicate an impairment of angiogenesis of these cells [137]. Conversely, CD105 negativity in human AT-MSC indicates a more efficient immunomodulatory capacity when compared to CD105-positive cells [140].

In line with the derangement observed in MSC-induced angiogenesis of rodents, AT-MSCs extracted from DM2 individuals with critical limb ischemia are dysfunctional, e.g., they exhibited a reduction in fibrinolytic activity and an increase in prothrombotic activity and PAI- levels. Those cells also possess lower efficiency of proliferation, migration, and CFU-f assay, as well as derangement in the PDGF (platelet-derived growth factor) signaling pathway [131]. PDGF signaling is known to modulate essential MSC processes, such as differentiation, migration, and proliferation, as well as coagulation and fibrinolysis systems. In addition, AT-MSC obtained from diabetic patients exhibited a decrease in angiogenic potential (lower level of VEGF expression and cell proliferation) when compared to healthy donors in a murine model of an ischemic flap [141]. Notably, VEGF and HGF secretion, tubulogenesis, and cell proliferation in diabetic conditioned media were increased in response to hypoxic stimuli, and it was similar to those of control cells. These findings may be important in the context of future study of autologous cell-based therapy in diabetic patients and indicate that hypoxia-mediated preconditioning may be a useful strategy for increasing the therapeutic potential of diabetic MSCs.

The change in the secretome of diabetic MSCs grown even under normoglycemic conditions is related to the development of metabolic memory, a process in which hypomethylation in gene promoters leads to dysregulation of gene expression and implies the persistence of DM-related complications even when glucose returns to normal levels. That effect is supported by studies that show changes in glucose metabolism in diabetic MSCs and by the fact that their functional capacities were not altered by normalization of glucose levels *in vitro* [120, 122].

Therefore, serum obtained from DM2 individuals may increase the BM-MSC proliferation *in vitro* rate, and HbA1c levels may play a role in that effect, indicating that higher rates of proliferation occur when HbA1c levels were 8-10% (versus HbA1c < 6.5%), yet serum derived from individuals with HbA1c > 10% exhibited a decrease in MSC proliferation [142]. On the other hand, diabetic serum decreased osteogenic differentiation in a concentration-dependent manner of HbA1c levels. These findings indicate the impact of the hyperglycemia control on MSC function and suggest that diabetic-derived MSC may be adversely affected in the diabetic milieu. A key aspect in that setting includes the adequate treatment of DM in order to support a better therapeutic potential of MSCs. Not only DM but also other chronic diseases, such as CKD, may impair MSC functionality. Autologous AT-MSCs obtained from CKD individuals (stages 3 and 4), when injected intravenously (1 × 10^6^/kg), exhibited a safety profile and contributed to decreasing proteinuria, yet not modifying eGFR in six patients [143]. Other progenitor cells, such as endothelial progenitor cells, are affected by uremia regardless of the presence of DM [144].

Notably, BM-MSC of newly diagnosed (<6 weeks) DM1 individuals (all males, 23.2 ± 2.9 years) presented similar morphology, immunophenotype, differentiation potential, gene expression of immunomodulatory molecules, and *in vitro* immunosuppressive capacity when compared to normal individuals [113]. However, the HGF gene was significantly downregulated in DM1-derived MSC. When injected into STZ-induced diabetic mice, both DM1 and control MSCs lead to improvement in serum glucose and insulin and in pancreatic histology.

In line with these findings, Davies et al. compared BM-MSC from individuals with newly diagnosed (<6 weeks) DM1 (*n* = 10; mean age 22 years, range 18-35 years; 9 males), late stage of DM1 with severe renal failure (*n* = 12, mean age 42 years, range 31-62 years; 7 males), and healthy BM donors (*n* = 19, mean age 37 years, range 21-70 years; 13 males) [136]. They found that gene expression was different between healthy controls and late DM1 in relation to cytokine secretion, immunomodulatory activity, and wound healing potential. Despite these difference between BM-MSC, DM1-derived MSCs did not demonstrate a significant difference from healthy controls in growth characteristics (CFU-f and doubling time), immunosuppressive activity, migratory capacity, or trophic properties at baseline and after exposure to proinflammatory cytokines IFN-*γ* and TNF-*α* (similar activity of IDO and upregulation of IL-6, CXCL1, and CXCL6).

To further substantiate the benefits of autologous MSC-based therapy, preconditioning strategies are key aspects to preserve MSC function, such as hypoxia culture, as previously described [141]. In addition, antioxidant pretreatment (N-acetylcysteine and ascorbic acid 2-phosphate) of BM-MSC from obese diabetic, B6.Cg-Lep^ob^/J mice significantly reduced the excessive TNF-*α* response observed in diabetic mice and improved IL-10 secretion [145]. Iron chelator deferoxamine pretreatment of human AT-MSCs increases hypoxia inducible factor 1-*α* (HIF-1*α*), which led to an upregulation of angiogenic factors (VEGF and angiopoietin-1), neuroprotective factors (nerve growth factor, glial cell-derived neurotrophic factor, and neurotrophin-3), and cytokines with anti-inflammatory activity (IL-4 and IL-5) [146]. Deferoxamine pretreatment also promoted the increase in the capacity of MSC secretome *in vitro*, which was associated with a decrease in neuron death. PDGF pretreatment of human AT-MSCs extracted from DM2 individuals rescued these cells from the diabetic phenotype by improving the proliferation, migration, and the capacity of clot lysing and repairing skin wound in an animal model [131].

Another approach to decrease abnormalities of BM-MSC obtained from DM1 and DM2 animals is the coculture with human umbilical cord extracts (Wharton's jelly extract supernatant). Therefore, Wharton's jelly extract supernatant represents a cocktail of growth factors (IGF-1, EGF, PDGF-AB, and b-FGF); components of extracellular matrixes (hyaluronic acid, collagen, and MUC-1), L-glutamate, and EXOs may also ameliorate proliferative capacity, motility, mitochondrial degeneration, endoplasmic reticular functions, and EXO secretion in both DM1- and DM2-derived BM-MSC, since that supernatant provide the physiological environment to preserve MSC properties and functionality [115]. These findings highlight the importance of seeking potential preconditioning approaches in the clinical setting. In addition, adenoviral transfection of Sirtuin3 in amniotic fluid stem cells protected these cells from high glucose-induced apoptosis by preserving mitochondrial function (increase in mitophagy, mitochondrial potential, respiratory function, and ATP levels, as well as a decrease in ROS, cytochrome c, and caspase activity) and ameliorating cell proliferation [118].

In conclusion, despite the fact that autologous MSC-based therapy has already been reported to ameliorate kidney injury, many difficulties must be overcome to successfully implement that therapy for treating DKD. Key aspects include the type of diabetes, time elapsed since the diagnosis due to cellular metabolic memory, and cell source, which may impair MSC functional properties.

In addition, some points are beyond the fact of choosing autologous or allogeneic MSCs for treating individuals with DM1 and DM2. Due to the expressive quantity of MSCs required to form a biobank and provide them to immediately infuse into patients, MSC expansion is a key aspect of cell therapy preparation. Both autologous and allogeneic MSCs cultured for a prolonged period may be affected by disturbance in the cellular structure and function. Chromosomal instability and aberrations have been shown in AT-MSCs after prolonged time *in vitro* [147], which leads to their discard. In contrast to these evidences, other researchers indicated MSC genetic stability during several passages in culture [148, 149]. Likewise, cell viability is another important characteristic to be assessed before administration, especially to avoid senescent cell infusion. Senescent cells have major alterations in the overall secretome components, leading to a switch from beneficial to a harmful profile [150].

Another important aspect that must be taken into account in cell therapy with MSCs is the fact that their beneficial effect can be neglected by the occurrence of adipogenic differentiation during long-term follow-up, which can contribute to glomerulosclerosis [78].

The malignant transformation of MSCs has not been described in clinical trials [151, 152]. As reviewed elsewhere, there are controversial data regarding protumorigenic effect of MSC on preclinical models. Some authors argued that MSCs are mobilized into the circulation with further migration and incorporation into the tumor microenvironment [153]. In that setting, MSC may contribute either to enhance tumor growth by decreasing apoptosis and promoting angiogenesis or to inhibit tumor growth in both in vitro and in vivo studies. Importantly, allogenic-derived MSC obtained from different sources and injected through different pathways for the treatment of broad clinical conditions, including graft-versus-host disease and cardiovascular and neurological diseases, was not associated with tumor development throughout a follow-up of 30 days to 6.8 years [153].

### 2.4. Clinical Studies

We have consulted the *Clinical Trials* web portal (*clinicaltrials.gov*, access in January 2020) with the keywords “mesenchymal stem cell” or “mesenchymal stromal cell” and “diabetes”. We defined inclusion criteria as completed studies that have reported results on PubMed. These studies were mainly single-center prospective phase I/II clinical trials, which evaluated safety and tolerability and explored the therapeutic effects of MSCs on beta-cell regeneration and the impact on fasting plasma glucose (FBG), HbA1c, endogenous insulin, and C-peptide increment and the reduction of daily insulin requirement ≥ 50%, which reached the efficacy level. A dose-escalating (0.3 × 10^6^/kg, 1.0 × 10^6^/kg, or 2.0 × 10^6^/kg) randomized-controlled trial assessing one intravenous infusion of MPCs (rexlemestrocel-L) in DM2 individuals without DKD documented safety and efficacy of cell therapy [154]. In patients treated with the highest dose, there was a significant decrease in HbA1c at 8 weeks with 33% of patients achieving the clinical target HbA1c < 7%.

To note, there was only one multicentric study, which also included individuals with DKD [155]. In that randomized (1 : 1 : 1), double-blind, sequential, dose-escalating (150 × 10^6^ or 300 × 10^6^, single intravenous dose), multicenter, and placebo-controlled trial, safety and efficacy of adult allogeneic BM-derived MPCs (MPCs, rexlemestrocel-L) were evaluated in type 2 diabetic individuals with DKD (eGFR 20-50 ml/min/1.73 m^2^). In terms of safety, no patients exhibit treatment-related severe adverse events and only one patient developed antibody specific to the donor HLA (antibody specificity to donor antigen (class I) B40; mean fluorescence intensity 530) at week 4 that were undetectable at week 12. The primary exploratory efficacy parameter comprised eGFR, so that the placebo-adjusted least square mean change in eGFR at week 12 was 4.4 ± 2.2 (p = 0.05) and 1.6 ± 2.2 ml/min/1.73 m^2^ (p = 0.47) for the 150 × 10^6^ and 300 × 10^6^ groups, respectively. Relative to placebo, there was a suggestion of stabilization of eGFR in the rexlemestrocel-L 150 × 10^6^ group, most notably at the 12-week primary endpoint. Importantly, when subgroup analyses were performed (GFR ≤ 30 or >30 ml/min/1.73 m^2^), the subgroup with eGFR > 30 ml/min/1.73 m^2^ treated with 150 × 10^6^ cells manifested a lower decrease in eGFR when compared to the control group at 12 weeks (*p* = 0.04). In addition, there was a statistically significant decrease in the median IL-6 values for the 300 × 10^6^ group compared to placebo at week 12, but not for other markers (HbA1c, TNF-*α*, and C-reactive protein).

We observed a balanced distribution between allogeneic-MSCs and autologous-MSC-based studies for both DM1 and DM2 individuals. MSCs have also shown beneficial effects on glycemic control when combined to hematopoietic stem cells (HSCs) or BM-MNCs. However, studies have still not been able to establish insulin-free status in this group of patients, even by differentiation of human AT-MSC into insulin-secreting MSCs (AT-ISC-MSC) [156–158]. That approach is based on growing MSCs with growth factors and serum with supplements, such as nicotinamide, activin A, exendin, pentagastrin, HGF, B-27, N2, and antibiotics for 4 days [158]. After that, these cells secrete C-peptide and insulin *in vitro* and express genes responsible for insulin secretion (pax-6, pdx1, and isl-1).

Likewise, a nonmyeloablative low-intensity conditioning regimen combined to MSC therapy failed to demonstrate insulin independence [156–158]. The objective of the treatment is to stop autoimmune destruction of *β*-cells with high-dose immunosuppressive drugs. A similar approach was also attempted to reset the deleterious immunologic system with a reconstituted one originated from autologous hematopoietic stem cells [159]. The rationale is to preserve residual *β*-cell mass and facilitate endogenous mechanisms of *β*-cell regeneration. For example, a nonmyeloablative low-intensity conditioning regimen combined to autologous AT-IS-MSC and HSC was based on rabbit antithymocyte globulin, methylprednisolone, and bortezomib [158]. For allogeneic AT-ISC-MSC associated with HSC infusion, nonmyeloablative low-intensity conditioning included target specific irradiation to subdiaphragmatic lymph nodes, spleen, part of pelvic bones, and lumbar vertebrae before cell infusion [156]. In addition, anti-T cell antibody (rabbit anti-thymocyte globulin) and anti-B cell antibody (ABA) were administered intravenously to prevent rejection and facilitate grafting of transplanted cells. Of importance, no immunosuppressive medication was required posttransplant. To note, the outcomes in *β*-cell function from those studies should also be analyzed in light of the use of the immunosuppressive regimen *per se*.

MSC-based therapy was considered a safe procedure in all studies that verified the therapeutic potential of these cells. In a systematic review and meta-analysis of clinical trials that evaluated MSC safety in more than a thousand individuals diagnosed with other clinical conditions, a significant association between MSC infusion and fever was shown [160]. However, no other immediate event (acute infusion toxicity), organ system complications, infection, and long-term adverse events (death, malignancy) were documented.

In terms of efficacy, both autologous- [157, 158, 161–164] and allogeneic- [155, 156, 165–168] derived MSCs accomplished the major secondary endpoints, as effective in changing metabolic hallmarks of DM, such as C-peptide synthesis and reducing exogenous insulin requirement, FBG, and HbA1c, as described in Tables 2 and 3, respectively. In the same way, allogeneic MSCs were effective as autologous MSCs in improving the final diastolic volume and left ventricular ejection fraction of patients with ischemic cardiomyopathy [169]. Notably, alloimmune reactions in those patients receiving allogeneic MSCs were very low (3.7%). In renal transplant patients, the infusion of both autologous [170] and allogeneic [171] MSCs was considered safe and effective. These data suggest the possibility of developing a biobank of allogeneic MSCs for therapeutic purposes in several pathologies, since these cells lack the expression of class II MHC (Major Histocompatibility Complex) antigens and costimulatory molecules (CD80/B7.1 and CD86/B7.2) [39]. Noteworthily, the potential impact of donor-to-donor heterogeneity and the potential immunogenicity of allogeneic cells, depending on the culturing conditions and passages, the microenvironment, and the differentiation state, may alter the immunogenic phenotype, as recently reviewed [172].

Importantly, MSC differentiation, when exposed to a proinflammatory microenvironment, may result not only in upregulation of cell surface immunogenic molecules but also in a decrease in immunoregulatory or immunosuppressive molecule secretion, such as PGE2, as reviewed elsewhere [173]. On the other hand, when MSCs differentiate into chondrocytes, they may not impair the production of immunomodulatory molecules. Therefore, some strategies may overcome immunogenicity, such as using 3D cell culture conditions and gene therapy [173]. Notwithstanding that controversial data, the formation of donor-specific antibodies after allogeneic MSC injection occurs eventually is not sustained and does not adversely affect the benefits of cell therapy in clinical practice [154, 155, 169, 173, 174]. However, the implications of the development of alloantibodies still need to be assessed over longer time periods, alongside the tolerability and efficacy of single and repeated administration of allogeneic MSC before definite conclusions can be established. As discussed elsewhere, some key aspects to be taken into account include both preclinical (e.g., increased vigilance of cellular immunity in preclinical experiments, development of strategies to reduce alloantigen expression on allo-MSCs, determination of optimal tissue source of MSC, combination of allo-MSC therapies with immunomodulatory drugs, replacement of highly immunogenic cells with alternatives, and optimizing the route of administration and culture conditions) and clinical (e.g., prescreening for antidonor responses, tracking the development of humoral immune responses, performance of functionally analyses of adaptive antidonor responses, and systemic public reporting results) approaches [173].

In addition, those studies also raised important questions regarding the protocols related to MSC source and viability, the number of infusions, the number of infused cells, routes of administration, the ability of MSCs to migrate to the injury site, the potency of the MSCs in the context of disease, model, and outcome measure [57]. A key aspect suggests that efficacy may be curtailed by the sequestration within the lungs and early elimination, as discussed previously in Preclinical Studies: Small and Large Animals.

In a meta-analysis of UCB cell-, UCB-MSC-, BM-MSC-, and HSC-based therapies for both DM1 and DM2 individuals (*n* = 22 studies), it was documented that almost 60% of DM1 individuals (*n* = 15 studies, 300 patients, including 40 controls) became insulin-independent for a mean period of 16 months after HSC-MSC treatment (mean dose of 6.99 ± 3.28 × 10^6^ cells/kg CD34^+^), as opposed to a negative response when these patients were treated with UCB cells (mean dose of 1.49 × 10^7^ nucleated cells; mean number of CD34^+^ cells was 1.26 × 10^6^) [175]. Likewise, UCB-MSC (range, 1.27 × 10^6^/kg to 1.88 × 10^7^/kg; mean dose 2.6 ± 1.2 × 10^7^) therapy was superior to BM-MSC (mean dose of 2.75 × 10^6^/kg) therapy for DM1 individuals, when C-peptide levels were compared, but not in terms of decreasing HbA1c. For DM2 individuals (*n* = 7 studies, 224 patients, including 92 controls), no conclusive recommendation was defined, yet DM-MNCs provided a better outcome when compared to UCB-MSCs in improving C-peptide levels and decreasing HbA1c. The administration of cell therapy early after DM diagnosis was more effective than intervention at later stages (relative risk = 2.0). To note, UCB cells (*n* = 3) and BM-MNCs (*n* = 107) were injected intrapancreatically, whereas UCB-MSCs (*n* = 22) were injected intravenously. In addition, mean doses of BM-MNCs were 17.29 × 10^8^ cells/kg (mean number of CD34^+^ cells was 3.15 × 10^6^) and mean doses of UCB cells were 5.29 × 10^9^ (mean number of CD34^+^ was 2.88 × 10^6^), whereas mean doses of UCB-MSCs were 1 × 10^6^/kg (either intravenous or intrapancreatic). Whether the number of injected cells and the route of injection (intrapancreatic versus intravenous) are associated with better outcomes, further studies are warranted to reach a definitive conclusion. Importantly, the patient clinical condition may also play a role in cell therapy, as diabetic ketoacidosis may impair its efficacy.

In another recent meta-analysis including DM2 individuals (*n* = 6 studies, 206 patients), treatment with autologous BM-MNCs (dose ranged from 382.6 ± 10^7^ to 2.8 ± 1.9 × 10^9^ cells) was effective in reducing HbA1c by 1.18% and insulin requirement during a follow-up of 12 months [176].

## 3. Future Directions

Strategies such as gene modification, optimization of culture conditions, and pretreatment conditioning may lead to an improvement in MSC functionality and a decrease in heterogeneity. These strategies comprise hypoxia culture, pharmacological agents, trophic factors/cytokines, small molecules, physical factors/materials, and gene modification, which may all contribute to better tissue repair and regeneration mediated by MSCs [177].

MSCs are generally grown in an environment with 21% oxygen tension. However, physiologically, MSCs are found in an environment with a much lower oxygen tension (1% to 7%). Thus, the cultivation or preconditioning of MSCs in a hypoxia environment with 2% or 5% oxygen allows these cells to remain multipotent and have greater proliferative and migratory capacity, in addition to lower senescence rates [178, 179]. Importantly, MSCs preconditioned by hypoxia do not differentiate into fibroblasts associated with tumors *in vitro* and do not induce tumors *in vivo* [178].

In order to reduce the heterogeneity of the MSC profile, which is defined by the different isolation and culture protocols, the preconditioning of these cells with proinflammatory factors has been the focus of investigation. Thus, the preconditioning of MSCs through stimulation with IFN-*γ*, TNF-*α*, PGE2, and NO oxide mitigated the heterogeneous behavior of MSCs on T lymphocyte proliferation assays and on late type hypersensitivity response [180].

MSCs can also be tested as carriers of genes or genetic modifications. Due to their ability to migrate to injury sites, MSCs represent a robust platform for delivery of genes associated with regeneration and repair of renal tissue, functioning as a “Trojan Horse” [181]. Thus, several genes associated with trophic factors may be used for these purposes, such as HGF and klotho, since they are renoprotective, as reviewed elsewhere [16].

In addition, genetic modifications of MSCs, which are also very promising in the context of DKD, include the overexpression of erythropoietin, CXCR4, CTLA4Ig, and IL-10/selectin, as well as the transfection of minicircles containing biological drugs, such as etanercept, which is a TNF-*α* blocker [182], and transfection of nanoparticles containing iron oxide, polymers, and plasmids [183].

Despite MSC-based preconditioning treatment that has not been associated with harmful effects, further studies are required to verify its effectiveness in maintaining MSC properties.

To advance MSC-based therapy, production of a large amount of these cells is challenging. Automated hollow-fiber bioreactors were validated to the development of large-scale manufacturing MSCs, providing cells with preserved characteristics and functionality when compared to the manual multilayer flask method [184, 185]. That approach may be cost- and time-saving at the end of the day.

MSC-derived secretome is a cell-free alternative for treating DM1 and DM2 individuals, which can bypass some issues related to autologous and allogeneic MSCs [186]. Some advantages include the absence of antigenic factors, time-saving obtainment, and the adaptation of MSC to produce preestablished secretome components, designed to target specific pathologies, even separating vesicles from soluble proteins and adapting cell product to each disease scenario.

## 4. Conclusions

Laboratory research set the basis for establishing further translation research including preclinical development and proof of concept in model systems. Thus, animal models indicate that syngeneic, autologous, allogeneic, and xenogeneic MSCs are effective for treating metabolic dysfunction in the DM setting and for halting the progression of DKD. MSCs demonstrated efficacy in controlling several biological processes, such as apoptosis, autophagy, fibrosis, inflammation, and oxidative stress, as well as in ameliorating renal functional and structural parameters.

BM-MSCs have been applied as the most valuable source of autologous cell transplants for diabetic complications in animals and humans. Despite differential gene expression, expanded MSCs from DM1 donors are phenotypically and functionally similar to healthy control MSCs with regard to their immunomodulatory, migratory potential, multilineage differentiation, and secretion of growth factors. MSCs from DM2 donors exhibit dysfunctional properties, such as senescence, angiogenesis impairment, higher rates of apoptosis, and lower clonogenic potential. Therefore, hyperglycemia may cause abnormalities in intrinsic BM-MSC, which might lose sufficient therapeutic effects in DM2 individuals. Of importance, DM2 donors are usually older and exhibit hyperglycemia for a longer duration, so that aging may play an additional role in MSC dysfunction. Therefore, preconditioning strategies can be used to recover the characteristics and functions of MSCs of diabetic patients before infusion and, thus, improve their performance in autologous therapies in terms of tissue repair and regeneration.

**Figure 1:**
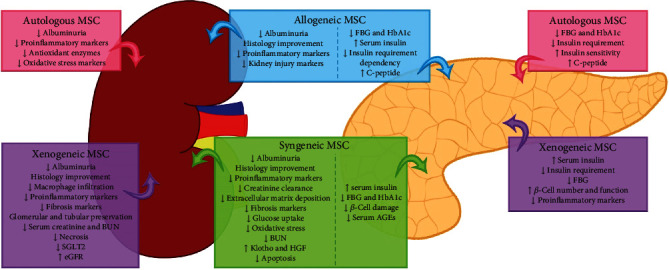
Main findings of preclinical and clinical studies evaluating MSC efficacy.

Together, the bench to bedside pathway has been constructed in the last decade. Laboratory research set the basis for establishing further translation research including preclinical development and proof of concept in model systems [187]. In Figure 1, we summarized the main findings of preclinical and clinical studies. Accordingly, phase I clinical trials (safety studies in humans) have substantiated the safe profile of MSC-based therapy, and phase II clinical trials (proof of concept in trial participants) still need to answer important questions. Therefore, well-designed large-scale randomized studies considering the stem cell type, cell number, and infusion method in DM patients are further needed in order to move to phase III clinical trials (large-scale trials to show significant efficacy). In addition, that pathway should include interactions with regulatory agencies and the protocols involved in the Investigational New Drug (IND) application development.

Clinical trials using MSC-based therapy indicate that infusion of autologous or allogeneic MSCs is generally well tolerated. Phase II clinical trials including a longer period of observation will support the efficacy of MSCs. However, the use of these cells to treat diabetic individuals with DKD awaits clinical validation. In conclusion, MSC-based preclinical and phase I/II clinical data encourage the design of future large-scale controlled clinical trials that evaluate DKD response to MSC therapy, while rigorous reporting of safety and efficacy is still needed.

## Figures and Tables

**Table 1:** Preclinical studies in small and large animals to verify the therapeutic potential of MSCs in DKD.

MSC type	MSC source	Model of DKD and groups	Number of injections/route of delivery	Number of cells injected	Results	Ref
Xenogeneic	h-BM	STZ-induced DM1 in NOD/*scid* mice: control, DKD, DKD+hMSC	Single dose, intracardiac	2.5 × 10^6^	DKD+hMSC versus DKD: ↑ Pancreatic insulin content and islet cell number ↓ Renal macrophage infiltration Improvement in renal histology	[98]
Syngeneic	BM	STZ-induced DM1 in C57BL/6 mice: DKD+vehicle and DKD+MSC	Single dose, IV	0.5 × 10^6^	DKD+MSCs versus DKD: ↓ FBG ↓ Albuminuria and glycosuria Improvement in renal and *β*-cell histology	[99]
Syngeneic	BM	STZ-induced DM1 in C57BL/6 mice: control, DKD+vehicle, DKD+MSC	Two doses (20 days apart), IV	0.5 × 10^6^	DKD+MSCs versus DKD: ↓ Albuminuria Improvement in renal histology No improvement in *β*-cell function and histology	[100]
Syngeneic	BM	STZ-induced DM1 in Sprague-Dawley rats: DKD, DKD+MSC, DKD+CSA, DKD+MSC+CSA (MSCA)	Single dose, intracardiac	2 × 10^6^	MSCA group versus DKD: ↓ FBG ↓ Albuminuria Improvement in renal mass index	[101]
Autologous	AT	STZ-induced DM1 in diabetes Sprague-Dawley rats: control, DKD+vehicle, DKD+AT-MSC	Single dose, IV	1 × 10^7^	DKD+AT-MSCs versus DKD: ↓ Renal p-p-38, p-ERK, and p-JNK ↓ Renal MDA and carbonyl protein ↓ Renal TNF-*α*, IL-1*β*, IL-6 ↓ Renal MnSOD and CuZn-SOD	[102]
Xenogeneic	h-UCB	STZ-induced DM1 in Sprague-Dawley rats: control, DKD, DKD+h-UCB-SC	Single dose, IV	1 × 10^6^	DKD+h-UCB-SCs versus DKD: ↓ FBG ↓ Albuminuria ↓ Renal fibronectin, *α*-SMA ↑ Renal E-cadherin	[103]
Xenogeneic	h-UCB	STZ-induced DM1 in Sprague-Dawley rats: control, DKD, DKD+h-UCB-SC	Single dose, IV	5 × 10^5^	DKD+h-UCB-SCs versus DKD: ↔ FBG ↔ Albuminuria Improvement in renal histology ↓ Renal TGF-*β*1, *α*-SMA ↑ Renal E-cadherin, BMP-7	[104]
Syngeneic	BM	STZ-induced DM1 in Sprague-Dawley rats: control, DKD+MSC, DKD+medium	Single dose, left renal artery	2 × 10^6^	DKD+MSCs versus DKD+medium: ↔ FBG ↓ Kidney weight, kidney/body weight, creatinine clearance ↓ Albuminuria Improvement in renal histology ↑ Renal nephrin, podocin, VEGF, BMP-7	[105]
Syngeneic, UTDM	BM	STZ-induced DM1 in Sprague-Dawley rats: control, DKD+vehicle, DKD+UTMD, DKD+MSC, DKD+MSC+UTMD	Single dose, IV	1 × 10^6^	DKD+MSC and DKD+MSC+UTMD versus DKD+vehicle and DKD+UTMD: ↓ FBG ↑ Plasma insulin Attenuated *β*-cell damage ↓ Albuminuria ↓ Renal TGF-*β*1 ↑ Renal synaptopodin, IL-10 ^∗^After UTMD: MSC homing was increased to kidneys (~2x)	[106]
Syngeneic	BM	STZ-induced DM1 in Wistar rats: control, DKD+vehicle, DKD+MSC	2 doses (1 week apart), IV	2 × 10^6^	DKD+MSCs versus DKD: ↓ FBG ↓ Albuminuria ↓ Creatinine clearance Improvement in renal histology ↓ Renal MCP-1, ED-1, IL-1*β*, IL-6, TNF-*α* ↑ Renal HGF	[107]
Syngeneic	BM	STZ-induced DM1 in Wistar rats: DKD, DKD+MSC, DKD+insulin, DKD+probucol	2 doses (1 week apart), IV	2 × 10^6^	DKD+MSCs versus DKD: ↓ FBG ↓ Albuminuria ↓ Creatinine clearance ↓ Kidney/body weight Improvement in renal histology ↓ Renal fibronectin, collagen I, TGF-*β*1, MDA content, ROS fluorescence ↑ Renal SOD activity ↓ Cellular glucose uptake mediated by GLUT1 in kidneys	[108]
Syngeneic	BM	STZ-induced DM1 in albino rats: control, DKD, DKD+vehicle, DKD+MSC	Single dose, IV	1 × 10^6^	DKD+MSCs versus DKD: ↓ FBG ↓ Albuminuria ↓ Body weight ↓ Serum creatinine and urea ↑ Renal VEGF and antiapoptotic bcl2 ↓ Renal TNF-*α*, proapoptotic Bax, TGF-*β* Improvement in renal histology	[109]
Syngeneic	BM	STZ-induced DM1 in Wistar rats: control, DKD+vehicle, DKD+MSC	2 doses (1 week apart), IV	2 × 10^6^	DKD+MSCs versus DKD: ↓ FBG ↓ Albuminuria ↓ Kidney/body weight ↓ Creatinine clearance Improvement in renal histology ↓ Renal collagen I, collagen IV, *α*-SMA, TGF-*β*, P-smad3/smad2/3 ↑ Renal E-cadherin, BMP-7	[47]
Syngeneic	BM ^∗^SDF-1-loaded microbubbles	STZ-induced DM1 in Sprague-Dawley rats: DKD+vehicle, DKD+UTMD, DKD+UTMD+MSC-SDF-1	Single dose, IV	1 × 10^6^	DKD+UTMD+MSC-SDF-1 versus DKD: Improvement in renal histology ↑ MSC engraftment with SDF-1 (7-fold versus control and 1.6-fold versus UTDM)	[110]
Syngeneic	BM	STZ-induced DM1 in C57BL/6 mice: DKD+vehicle, DKD+MSC	Single dose, IV	0.5 × 10^6^	DKD+MSCs versus DKD: ↓ Kidney ↓ Kidney/body weight ↓ Serum creatinine, urea, and plasma cystatin C ↓ Renal collagen I and fibronectin ↓ Renal tubular apoptotic index, ROS total, lipid peroxidation, oxidative protein damage, F4/80-positive cells ↑ Renal nephrin, tubular Ki67 proliferation index ↑ Plasma bFGF, EGF, HGF, IL-6, and IL-10 Improvement in renal histology	[111]
Syngeneic	AT	STZ-induced DM1 in Sprague-Dawley rats: control, DKD, DKD+vehicle, DKD+MSC	Single dose, IV	1 × 10^7^	DKD+MSCs versus DKD: Improvement in renal histology ↓ Kidney apoptosis (TUNEL, ↓ Bax and ↑ Bcl2), expression of Wnt1, Wnt3a, Snail, active *β*-catenin ↑ Renal klotho	[49]
Syngeneic	BM	STZ-induced DM1 in Sprague-Dawley rats: control, DKD, DKD+MSC	Single dose, IV	2 × 10^6^	DKD+MSC versus DKD: ↔ FBG ↓ Albuminuria ↓ Kidney weight ↓ Serum creatinine ↓ Renal PAI-1, TGF-*β*1, Smad3	[112]
Xenogeneic	h-BM (DM1 and normal individuals)	STZ-induced DM1 in C57BL/6 mice: DKD+DM1-MSC, DKD+control MSC, DKD+vehicle	Single dose, intrasplenic	1 × 10^6^	DKD+MSC versus DKD: ↓ FBG (~70% of mice) ↑ Serum insulin Improvement in glucose tolerance test Improvement in pancreatic inflammation (↓ IL-2 and INF-*γ*) and *β*-cell function	[113]
Xenogeneic (Lewis and SD-Tg rats -> C57BL/6J and C57BL/6-Tg mice)	BM	STZ-induced DM1 and HFD-induced DM2 in C57BL/6J and C57BL/6-Tg mice: Control, STZ+vehicle, STZ+MSC, STZ+MSC-CM Control, HFD+vehicle, HFD+MSC, HFD+MSC-CM	STZ model: 2 doses (4 weeks apart) HFD model: 4 doses (2 weeks apart) IV	1 × 10^4^ MSC/body weight	STZ model: STZ+MSC and STZ+CM-MSC versus STZ+vehicle Improvement in renal histology ↓ FBG: all groups versus control ↓ Renal TNF-*α*, ICAM-1, p-p38-MAPK ↑ Renal ZO-1, megalin HFD model: HFD+MSC and HFD+CM-MSC versus HFD+vehicle Improvement in renal histology ↓FBG: all groups versus control; HFD-MSC versus HFD-vehicle ↓ Renal TNF-*α*, ICAM-1, TGF-*β* ↑ Renal ZO-1, megalin	[50]
Syngeneic	BM	STZ-induced DM1 in albino Wistar rats: control, DM, DKD, DM+MSC, DKD+MSC	Single dose, IV	1 × 10^6^	MSC-treated versus nontreated: ↓ Serum creatinine, urea, uric acid ↓FBG ↑ Serum insulin ↓ Albuminuria ↓ Serum TGF-*β*, FGF-2, PDGF ↔ Serum AGEs ↑ Serum HO-1 activity ↓ Renal IL-8, MCP-1	[114]
Syngeneic (from each model of diabetic and control rats)	BM+treatment with UCB extracts preinfusion	STZ-induced DM1 in Sprague-Dawley rats and C57BL/6 mice; DM2 in OLETF diabetic rats: control, STZ or OLEFT, STZ+MSC, OLEFT+MSC	Four doses (2 weeks apart), IV	1 × 10^4^ MSC/body weight	MSC-treated versus nontreated: ↔ FBG ↔ Albuminuria ↔ Renal histology MSC+UCB extract-treated versus nontreated: ↔ FBG ↓ Albuminuria Improvement in renal histology	[115]
Syngeneic	BM+treatment with melatonin preinfusion	STZ-induced DM1 in Wistar rats: control, DKD, DKD+MSC, DKD+MSC+melatonin	Single dose, IV	1 × 10^6^	DKD+MSCs versus DKD (effects intensified with melatonin): ↑ Renal SOD, Beclin-1 ↓ Renal TGF-*β*	[116]
Syngeneic	BM	STZ-induced DM1 in Sprague-Dawley rats: control, DKD+vehicle, DKD+MSC	Four doses (1-2 weeks apart), IV	5 × 10^6^	DKD+MSCs versus DKD: ↑ Rat survival ↓ Serum urea ↓ Albuminuria ↓ Renal TGF-*β*1, fibronectin, ICAM-1, MCP-1, CD68, TNF-*α*, IL-6, IL-1*β* ↓ Serum IL-1*α*, IL-1*β*, IL-6, IFN*γ* Improvement in renal histology	[52]
Syngeneic	BM+transfection with miR-124a	STZ-induced DM1 in Sprague-Dawley rats: control, MSC, DKD, DKD+MSC with miR124a mimics, inhibitors, and negative control	Single dose, IV	3 × 10^6^	DKD+MSCs versus DKD: ↔ FBG ↔ Albuminuria MSC+miR124a: ↑ Renal nephrin, podocin, CD2AP, Bcl-2 ↓ Renal TGF-*β*1, collagen I and III, caspase-3, Bax	[45]
Xenogeneic (human -> monkeys)	BM	STZ-induced DM1 in cynomolgus monkeys (*Macaca fascicularis*) treated with insulin glargine and glulisine+acute ischemia-reperfusion injury: control, DKD, DKD+MSC	Single dose; intra-arterial (suprarenal aorta)	5 × 10^6^ cells/kg	DKD+MSCs versus DKD: ↔ Serum creatinine, urea, TNF-*α*, IFN-*γ* ↔ Albuminuria ↔ Urinary NGAL, GST-*α*, and TIMP-1 Improvement in renal histology (↓ necrosis)	[117]
Syngeneic	Amniotic liquid (adenovirus SIRT3 overexpression)	db/db mice: wild type, control, DKD+adenovirus control, DKD+adenovirus-SIRT3	Single dose, intraparenchymal	3 × 10^6^	DKD versus DKD+SIRT3: ↓ Body weight ↓ FBG, serum insulin, C-peptide, glucagon, HbA1c ↓ Serum creatinine, urea ↓ Serum TNF-*α*, IL-6, MCP-1 ↓ Systolic blood pressure ↓ Albuminuria ↓ Kidney weight, oxidative stress, collagen I/III/IV deposition, MMP9, TGF-*β* Improvement in renal histology	[118]
Syngeneic	BM	STZ-induced DM1 in Sprague-Dawley rats: control, DKD+vehicle, DKD+MSC, DKD+MSC+WRW4 (1 mg/kg), DKD+LXA4 (10 mg/kg), DKD+LXA4+WRW4	Two doses (1 week apart), IV	5 × 10^6^	DKD+MSCs versus DKD: ↑ Rat survival ↔ FBG ↓ Serum creatinine and urea ↓ Glycosuria, albuminuria ↑ Renal LXA4 ↓ Renal TGF-*β*1, p-SMAD2/3 ↓ Serum TNF-*α*, IL-6, IL-8, IFN-*γ* (LXA4 treatment exhibited similar findings when compared to MSC, which was abrogated by WRW4 treatment)	[48]
Allogeneic (from CAG-EGFP.SD-Tg rats)	AT	Spontaneously diabetic Torii (SDT) fatty rats (SDT.Cg-Lepfa/JttJcl)+unilateral nephrectomy: control; DKD+MSC suspension via IV route; DKD+MSC sheets transplanted directly into the kidney	Single dose, IV or cell sheets transplanted directly into the kidney	6 × 10^6^/ml via IV route and cell sheets	DKD+cell sheets versus DKD+MSC via IV route and DKD: ↓ Albuminuria, proteinuria, and urinary L-FABP, KIM-1, IL-6 Improvement in renal histology DKD+cell sheets and DKD+MSC via IV route versus DKD: ↓ Urinary podocalyxin and TNF-*α*	[90]
Xenogeneic (human -> macaques)	UCB	STZ-induced DM1 in rhesus macaques+high-fat and high-salt diet (for 2 years): control; DKD; DKD+MSC	4 doses (2 weeks apart), IV	2 × 10^6^/kg	DKD+MSCs versus DKD: ↓ FBG, insulin requirement ↓ Serum creatinine and BUN ↑ eGFR ↓ Albuminuria ↓ Renal IL-1*β*, IL-16, TNF-*α*, CTGF, SGLT2 ↑ Renal IL-6 ↓ Serum IFN-*γ*, TNF-*α*, IL-1*β*, IL-5, IL-12p70, IL-15, IL-16 Improvement in renal histology	[44]
Xenogeneic (human -> mice)	UCB	Unilateral nephrectomy+STZ-induced DM1 in CD1 mice	3 doses (4 weeks apart), IV	5 × 10^5^	DKD+MSCs versus DKD: ↔ Serum glucose ↓ Serum creatinine and BUN ↓ Albuminuria ↓ Renal mRNA desmim, *α*-SMA, Fn1, Kim-1, NGAL, MCP-1, VCAM-1, ICAM-1, IL-1b, TNF-*α*, IL-6, iNOS ↑ Renal mRNA arginine 1 Improvement in renal histology	[53]

MSCs: mesenchymal stem cells; BM-MSC: bone marrow-derived MSCs; h-BM-MSC: human bone marrow-derived MSC; AT-MSC: adipose tissue-derived MSCs; h-UCB-SCs: human umbilical cord blood-derived stem cells; MSC-CM: MSC-conditioned medium; DM: diabetes mellitus; DKD: diabetic kidney disease; AGEs: advanced glycation end products; BMP-7: bone morphogenic protein-7; CSA: cyclosporine; EGF: epidermal growth factor; FBG: fasting blood glucose; bFGF: basic fibroblast growth factor; Fn1: fibronectin-1; GST-*α*: glutathione S-transferase-*α*; HFD: high-fat diet; HGF: hepatocyte growth factor; HO-1: heme-oxygenase-1; ICAM-1: intercellular adhesion molecule-1; iNOS: inducible nitric oxide synthase; IL: interleukin; IFN-*γ*: interferon-*γ*; IV: intravenous; KIM-1: kidney injury molecule-1; LETO: Long-Evans Tokushima Otsuka rats; L-FABP: liver-type fatty acid binding protein; LXA4: lipoxin A4; MDA: malondialdehyde; miR: microRNA; MCP-1: monocyte chemoattractant protein-1; MMP: matrix metalloproteinase; NGAL: neutrophil-gelatinase associated lipocalin; OLETF: Otsuka Long-Evans Tokushima Fatty diabetic rats; PAI-1: plasminogen activator inhibitor-1; PDGF: platelet-derived growth factor; ROS: reactive oxygen species; SDF-1: stromal-derived factor-1; SIRT3: sirtuin 3; SOD: superoxide dismutase; *α*-SMA: *α*-smooth muscle actin; STZ: streptozotocin; TGF-*α*: transforming growth factor *α*; TGF-*β*1: transforming growth factor *β*1; TIMP-1: tissue inhibitor metalloproteinase-1; TNF-*α*: tumor necrosis factor-*α*; UTMD: ultrasound-targeted microbubble destruction; VCAM-1: vascular cell adhesion molecule-1; VEGF: vascular endothelial growth factor; ZO-1: *zonula occludens*-1.

**Table 2:** Autologous MSC-based clinical trials.

MSC source	DM type	Age (years)	Time of DM	Number of patients	Number of injections/route of delivery	Number of cells injected	Follow-up (m)	Results	Adverse events	Ref
BM	1	<8 y	<2 m	2	Single dose, liver	180 × 10^6^/kg	12	↑ C-peptide ↓ FBG, HbA1c Negative values of ICA, GAD and anti-insulin antibody levels	None	[161]
BM	1	18-40 y	<3 w	10 treated with insulin and 10 treated with insulin+MSCs	Single dose, IV	2.1-3.6 × 10^6^/kg (median 2.75 × 10^6^/kg)	12	↑ C-peptide after mixed-meal tolerance test ↔ HbA1c, insulin dose, fasting C-peptide	None	[162]
AT-ISC-MSC+BM-HSC ^∗^conditioning	1	Group 1: 20 ± 7 y Group 2: 20 ± 10 y	Group 1: 8.1 ± 3.4 y Group 2: 9.9 ± 7.1 y	Group 1: autologous AT-ISC-MSC+BM-HSC Group 2: allogeneic AT-ISC-MSC+BM-HSC from healthy nondiabetic donors (*n* = 10 each)	Single dose, portal+intrathymus+SC	Group 1: AT-ISC-MSC-2.65 ± 0.8 × 10^4^/kg Group 2: AT-ISC-MSC-2.07 ± 0.67 × 10^4^/kg	24	Autologous versus allogeneic: (i) Allogeneic: ↓ insulin requirement up to 6 months and ↓ postprandial blood sugar from months 1-24 (ii) Autologous: ↑ C-peptide from months 15-24 (iii) Allogeneic = autologous: ↓ HbA1c, FBG, GAD antibody	None	[157, 158]
BM	2	30-60 y	≥5 y	BM-MSC versus BM-MNCs versus control group (antidiabetic drugs) (*n* = 10 each)	Single dose, superior pancreatic-duodenal artery	MSCs: 1 × 10^6^/kg MNCs: 1 × 10^7^	12	↓ Insulin requirement ≥ 50% and HbA1c in 60% of individuals (BM-MSC and BM-MNC) ↔ HOMA indexes ↑ Insulin sensitivity index+insulin receptor substrate-1 gene expression in muscle (BM-MSC) ↑ Second phase C-peptide response during hyperglycaemic clamp (BM-MNCs)	Nausea and vomiting (*n* = 2)	[163]
BM	2	37-67 y (52.9 y)	2-15 y (7.15 y)	NPR (*n* = 19) PR (*n* = 15)	Single dose, IV	3.0 × 10^6^/kg	6	↓ FBG, hypersensitivity C-reactive protein ↔ HbA1c, plasma IL-6 ↓ Macular thickness and improvement in best corrected visual acuity (proliferative retinopathy group)	None	[164]

AT-ISC-MSC: adipose-derived insulin-secreting mesenchymal stem cells; BM-HSCs: bone marrow-derived hematopoietic stem cells; BM-MNCs: bone marrow-derived mononuclear cells. Autoantibodies to *β*-cells: GAD65 (glutamic acid decarboxylase) and IA2 antibodies. FBG: fasting blood glucose; HbA1c: glycated haemoglobin; NPR: nonproliferative retinopathy; PR: proliferative retinopathy; SC: subcutaneous; IU: International Units; IV: intravenous; w: week; m: month; y: years.

**Table 3:** Allogeneic MSC-based clinical trials.

MSC source	DM type	Age	Time of DM	Number of patients	Number of injections/route of delivery	Number of cells injected	Follow-up (m)	Results	Adverse events	Ref
AT-ISC-MSC+HSC ^∗^Conditioning	1	13-43 y (21 y)	1-24 y (8.2 y)	11	Single dose, omental vein	Mean total cell quantum transplanted: 96.3 ml (92-118 ml) HSC: 28 × 10^3^/*μ*l (12.2- 62.7 × 10^3^/*μ*l) MSC: 1.2 × 10^3^/*μ*l (0.5-2.1 × 10^3^/*μ*l)	23	↓ Insulin requirement, HbA1C ↑C-peptide	None	[156]
Placental	2	45-82 y (66 y)	3-20 y (11 y)	10	Single dose, IV	1.35 × 10^6^/kg (1.22-1.51 × 10^6^/kg)	3	↓ Insulin requirement, HbA1c ↑ C-peptide, insulin	None	[165]
Wharton's jelly-MSC	1	Group 1 (MSC): 17.6 ± 8.7 y Group 2 (saline): 18.2 ± 7.9 y	Newly onset	29 Group 1: *n* = 15 Group 2: *n* = 14	Two doses (4 weeks apart), IV	2.6 ± 1.2 × 10^7^ (1.5-3.2 × 10^7^)	21	↔ FBG, GAD antibody ↓ Insulin requirement, postprandial glucose, HbA1c ↑ C-peptide, C-peptide/glucose ratio	None	[166]
Wharton's jelly-MSC	2	52.9 ± 10.5 y	8.7 ± 4.3 y	22	One dose IV and one dose intrapancreatic	1 × 10^6^/kg	12	↓ FBG, postprandial glucose, HbA1c, insulin requirement ↑ C-peptide, HOMA-*β* ↓ Serum CD3^+^ and CD4^+^ lymphocytes ↔ Serum CD8^+^ lymphocytes ↓ Serum IL-6, IL-1*β* ↔ Serum TNF-*α* and IL-10	Fever (*n* = 3), hematoma (*n* = 1), nausea, vomiting, headache (*n* = 1)	[167]
BM (rexlemestrocel-L) ^∗^eGFR: 20-50 ml/min/1.73 m^2^	2	Placebo: 74.8 ± 7.9 y Lower dose MSCs: 70.5 ± 7.4 y Higher dose MSCs: 64.8 ± 10.1 y	Time of DM: N/A	30 (*n* = 10 each)	Single dose, IV	150 × 10^6^/kg (lower dose) or 300 × 10^6^/kg (higher dose)	12	↔ eGFR, albuminuria ↔ Lipid profile ↔ Blood pressure ↔ Serum C-reactive protein, TNF-*α* ↓ Serum IL-6	None	[155]
UC+autologous BM-MNC	1	Standard care: 13-27 y (20.4 y) Cell therapy: 5-28 y (18.3 y)	Standard care: 2-13 y (7.0 y) Cell therapy: 2-16 y (9.2 y)	42 (*n* = 21 each)	Single dose of each cell, dorsal pancreatic artery	UCB: 1.1 × 10^6^/kg BM-MNCs: 106.8 × 10^6^/kg	12	Cell therapy versus standard care: ↑ AUC of C-peptide ↑ AUC of insulin ↓ FBG, HbA1c ↓ Fasting C-peptide ↓ Insulin requirement ↓ Anxiety score ↓ Depression score ↑ Quality of life	Upper respiratory tract infection (*n* = 7), bleeding (*n* = 1), abdominal pain (*n* = 1)	[168]

AT-ISC-MSC: adipose-derived insulin-secreting mesenchymal stem cells; AUC: area under the curve; BM-HSCs: bone marrow-derived hematopoietic stem cells; BM-MNCs: bone marrow-derived mononuclear cells; FBG: fasting blood glucose; eGFR: estimated glomerular filtration rate; HbA1c: glycated haemoglobin; IV: intravenous; IU: International Units; m: month; NPR: nonproliferative retinopathy; PR: proliferative retinopathy; SC: subcutaneous; ZO-1: *zonula occludens-1*; y: years.

## Data Availability

Our manuscript is based on a review of articles that have been already published on PubMed.

## Abbreviations

AGEs: Advanced glycation end products
AMDCC: Animal Models of Diabetic Complications Consortium
AT-MSC: Adipose tissue-derived MSCs
AT-IS-MSC: AT-insulin-secreting-derived MSCs
BM-MSC: Bone marrow-derived MSCs
BTBR*^ob/ob^* mice: Black and tan, obese, and tufted *ob/ob* (leptin deficient) mice
CFU-f: Colony Forming Unit-fibroblast
CKD: Chronic kidney disease
CV: Cardiovascular
CXCL: Chemokine (C-X-C motif) ligand
CXCR: C-X-C chemokine receptor
DKD: Diabetic kidney disease
DM: Diabetes mellitus
DPP-4 inhibitors: Dipeptidyl peptidase-4 inhibitors
DRD: Diabetic renal disease
ESKD: End-stage kidney renal disease
EXOs: Exosomes
FBG: Fasting plasma glucose
GBM: Glomerular basement membrane
eGFR: Estimated glomerular filtration rate
GLP1-RA: Glucagon-like peptide 1 receptor agonists
GMCs: Glomerular mesangial cells
GMP: Good Manufacturing Practice
HbA1c: Glycated haemoglobin
HGF: Hepatocyte growth factor
HSCs: Hematopoietic stem cells
IDO: Indoleamine 2,3-dioxygenase
IFN-*γ*: Interferon-*γ*
IGF-1: Insulin growth factor
IL: Interleukin
IND: Investigational New Drug
iNOS: Inducible nitric oxide synthase
ISC: Insulin-secreting cells
ISCT: International Society for Cell Therapy
LPS: Lipopolysaccharide
MCP-1: Monocyte chemoattractant protein-1
MHC: Major complex of histocompatibility
MIP: Macrophage inflammatory protein
MSCs: Mesenchymal stem/stromal cells
NO: Nitric oxide
NOD: Nonobese diabetic mouse
PDGF: Platelet-derived growth factor
PGE2: Prostaglandin E2
PKC: Protein kinase C
RAAS: Renin-angiotensin-aldosterone system
Sca-1: Stem cell antigen-1
SDF-1: Stromal-derived factor-1
SGLT(i): Sodium-glucose cotransporter (inhibitors)
STZ: Streptozotocin
TGF-*β*1: Transforming growth factor-*β*1
TLR: Toll-like receptors
TNF-*α*: Tumor necrosis factor-*α*
TEC: Tubular epithelial cells
UAE: Urinary albumin excretion
UCB-MSCs: Umbilical cord blood-derived MSCs
UTMD: Ultrasound-targeted microbubble destruction
VEGF-A: Vascular endothelial growth factor A.
